# Autologous decellularized extracellular matrix protects against H_2_O_2_-induced senescence and aging in adipose-derived stem cells and stimulates proliferation *in vitro*

**DOI:** 10.1042/BSR20182137

**Published:** 2019-05-21

**Authors:** Xiaofang Yu, Yucang He, Zhuojie Chen, Yao Qian, Jingping Wang, Ziwan Ji, Xiaoyan Tan, Liqun Li, Ming Lin

**Affiliations:** 1 Second Affiliated Hospital and Yuying Children’s Hospital of Wenzhou Medical University, West Xueyuan Road, Lucheng Direct, Wenzhou City, Zhejiang Province, People’s Republic of China; 2 First Affiliated Hospital of Wenzhou Medical University, Nanbaixiang, Ouhai Direct, Wenzhou City, Zhejiang Province, People’s Republic of China; 3 Hangzhou plastic surgery hospital, Xiacheng Direct, Hangzhou City, Zhejiang Province, People’s Republic of China

**Keywords:** Adipose-derived stem cells, extracellular matrix, hydrogen peroxide, lipotransfer, multipotency, senescence

## Abstract

**Background:** Adipose-derived stem cells have attracted significant interest, especially in stem cell therapy and regenerative medicine. However, these cells undergo gradual premature senescence in long-term cultures, which are essential for clinical applications that require cell-assisted lipotransfer or tissue repair. **Methods:** Since the extracellular matrix forms the microenvironment around stem cells *in vitro* and regulates self-renewal and multipotency in part by slowing down stem cell aging, we evaluated its potential to protect against senescence, using H_2_O_2_-induced adipose-derived stem cells as a model. **Results:** We found that supplementing cultures with decellularized extracellular matrix harvested from the same cells significantly promotes proliferation and reverses signs of senescence, including decreased multipotency, increased expression of senescence-associated β-galactosidase, and accumulation of reactive oxygen species. **Conclusion:** These findings suggest a novel approach in which an autologous decellularized extracellular matrix is used to prevent cellular senescence to enable the use of adipose-derived stem cells in regenerative medicine.

## Introduction

In 2001, Zuk et al. [1] first isolated adipose-derived stem cells from lipoaspirate, and showed that these cells were capable of differentiating into adipocytes, osteoblasts, and chondroblasts. In addition, these cells were abundant in the source tissue and were easy to isolate, and culture, and thus are ideal as seed cells for regenerative medicine and tissue engineering, such as improving functional recovery of skeletal muscle [2] and promoting the survival of fat grafts [3]. Unfortunately, the replicative cycle is very long both *in vitro* and *in vivo*, so attempts to use these cells must overcome the inevitable senescence, loss of stemness and self-healing, and diminished proliferation and differentiation. In addition, aging cells gradually trigger a series of reactions, including apoptosis and autophagy, which may stress the whole organism [4]. These limitations clearly illustrate the need to develop cell culture conditions that enable slow aging of the adipose-derived stem cells.

Adipose-stem cells are surrounded by extracellular matrix, which is a fibrous material coated with an array of molecules secreted by cells [5]. The autologous extracellular matrix provides a net-like structure that supports and connects many stem cells, and that regulates intercellular communication, adhesion, migration, and proliferation [6,7] via an array of cellular factors stored in it [8]. It is also a quintessential component of cellular niches in tissues by supplying critical biochemical and physical cues that initiate or sustain cellular functions.

During culture *in vitro*, cells except hemocytes produce their own extracellular matrix, which can then be decellularized and purified. For example, Lai et al. [9] reconstituted marrow-derived extracellular matrix, and demonstrated that it enables large-scale expansion of highly functional mesenchymal stem cells suitable for therapy. Another study unraveled a new mechanism by which the extracellular matrix regulates endothelial cell function altering redox balance as an essential component of redox-signaling [10]. However, few studies have examined the decellularized extracellular matrix function on adipose-derived stem cells.

Accordingly, we proposed an alternative approach in which an autologous decellularized extracellular matrix is produced from adipose-derived stem cells *in vitro* and then used to culture the same cells. Our objective was to test the possibility that the autologous decellularized extracellular matrix slows senescence- and aging-related phenomena in H_2_O_2_-accelerated aging adipose-derived stem cells, or as part of the microenvironment, promote cell proliferation *in vitro*.

## Materials and methods

### Source of human fat tissues

Human adipose tissue was obtained solely from tissues collected by liposuction from five healthy adult female or male patients (range, 22–34 years). All patients provided written informed consent for research use of tissues and for publication. Ethical approval was also obtained from the Research Ethics Committee of Wenzhou Medical University.

### Reagents and catalog numbers

0.25% Collagenase I and H_2_O_2_ were purchased from Sigma (C0130, 88597). Human adipose-derived stem cell growth medium, mesenchymal stem cell adipogenic differentiation medium, and mesenchymal stem cell osteogenic differentiation medium were obtained from Cyagen (HUXMD-90011, GUXMX-90031, GUXMX-90021). Triton X-100, NH_4_OH, 4% formaldehyde, and Reactive Oxygen Species Assay Kit were purchased from Solarbio Science (T8200, G1820, P1110, CA1410). DNase I was purchased from Takara (2270A). Cell-Light Edu Apollp567 In Vitro Kit was purchased from RiboBio (C10310). Cell Counting Kit-8 was obtained from Dojindo (CK04). Senescence β-Galactosidase Staining Kit was purchased from Beyotime (C0602). All other chemicals used were of the highest grade commercially available.

### Isolation and collection of human adipose-derived stem cells

Adipose tissues were drained of blood, and then digested for 45 min at 37°C by shaking in an equal volume of 0.25% Collagenase I (Sigma, USA). Digestion was terminated by adding complete culture media consisting of Dulbecco’s modified Eagle’s medium, 10% fetal bovine serum, and 0.1% penicillin (Cyagen Biosciences, Guangzhou, China). Undigested tissues and unneeded oils were filtered out using a screen mesh, and the resulting cell suspension was centrifuged for 5 min at 1200 rpm. The pellet was then immediately resuspended in fresh complete medium and recentrifuged. Finally, cells were incubated at 37°C and 5% CO_2_, and imaged after 2 or 3 days on an Olympus CKX41 microscope.

### Preparation of cell-free extracellular matrix

Third passage adipose-derived stem cells were seeded onto 12-well plates (Corning, NY, USA) at 1 × 10^5^ cells/well in 1 ml complete culture media and sealed with a 20-mm microscope coverslip (NEST Biotechnology, Wuxi, China). Cells were cultured to 60–70% confluence over approximately 2 days, at which point culture media were replaced with media containing 50 μM l-ascorbic acid (MedchemExpress, U.S.A.) to induce film formation over another 8 days. Media were changed every 3–4 days in this duration. For further decellularization, cells were extensively washed in PBS (phosphate buffer saline), and the extracellular matrix was obtained by incubating for 5 min at 37°C in PBS with 0.5% Triton X-100 (Solarbio Science, Beijing, China) and 20 mM NH_4_OH (Solarbio Science, Beijing, China) [7]. Cells and cell debris were then removed, leaving only the extracellular matrix, which was subsequently incubated with 100 U/ml DNase I (Takara, Japan) for 1 h at 37°C. Samples were then carefully washed three times with PBS to remove residual DNase I, and stored sterile at 4°C [11].

### Scanning electron microscopy

Samples before and after decellularization were both fixed in 2.5% glutaraldehyde overnight and dehydrated through a gradient of 50–100% ethanol. Specimens were then purged with tertiary butyl alcohol, dried in a VFD-21S *t*-BuOH freeze dryer (China), coated with gold–palladium, and imaged on a HITACHI S-3000N scanning electron microscope (Japan) to analyze morphology and microstructure.

### Expansion of adipose-derived stem cells on autologous extracellular matrix

After three passages, adipose-derived stem cells were inoculated at 3 × 10^5^/well in six-well plates with shaking, half of which contained the decellularized extracellular matrix substrates harvested as described above. After 3 days, an inverted microscope was used to observe and image the morphology of the adipose-derived stem cells. Afterwards, the Image J software was used to estimate the numbers of adipose-derived stem cells seeded *on autologous extracellular matrix* by measuring the percentage of areas covered by adherent cells in each picture (*n* = 10).

### Edu staining

DNA replication and cell proliferation were assessed using Cell-Light Edu Apollp567 *In Vitro* Kit (RiboBio, Guangzhou, China). Briefly, the decellularized extracellular matrix was placed in 12-well plates and seeded with 5 × 10^4^ adipose-derived stem cells/well. At sub-confluence 2 days later, cells were incubated for 2 h with 1:1000 Edu, and fixed using 4% formaldehyde (Solarbio Science, Beijing, China). After further washes, cells were successively stained with Apollo and DNA staining in the dark, and imaged on an ECLIPSE TI fluorescence inverted microscope (Nikon, Japan). Cells grown without decellularized extracellular matrix were used as a control.

### Treatment with H_2_O_2_

Passage three cells at logarithmic phase were seeded at 1 × 10^4^ cells/well in 20 wells in each of five 96-well plates. Almost all cells adhered within 1 day, and five wells each in one plate were exposed for 2 h to 50, 100, or 150 μM H_2_O_2_ (Sigma–Aldrich, St. Louis, MO, USA). H_2_O_2_ was then removed by gently washing twice in PBS (Gibco, NY, USA), and cells were incubated for another 4 h in complete media, stained with 10 μl Cell Counting Kit-8 reagent (Dojindo, Kumamoto, Japan) for 1–4 h at 37°C, and assayed at 480 nm using a SPECTRA max PLUS 384 microplate reader (U.S.A.). The other four plates were assayed in the same manner every 24 h on subsequent days. Cell viability was measured as fold-change in absorbance at 480 nm in comparison with the absorbance of cells not exposed to H_2_O_2_.

### Staining for senescence-associated β-galactosidase

Senescence-associated β-galactosidase, a typical biomarker of premature cellular senescence, was quantified in adipose-derived stem cells exposed to fresh complete media for an additional 52 h after inducted by 100 μM H_2_O_2_ for 2 h. The cells were then fixed with 4% paraformaldehyde for 30 min at room temperature, washed with PBS three times, and stained overnight at 37°C in the dark and without CO_2_ using a staining kit (Beyotime Institute of Biotechnology, Haimen, China), following the manufacturer’s instructions. The staining buffer was removed the following day, and cells were washed with PBS three times and imaged on an Olympus CKX41 microscope. Senescent cells are stained green in this assay. The senescence-associated β-galactosidase positive cells were quantified in five random fields per dish and a total of at least 200 cells from each sample were counted per field. Senescence-associated β-galactosidase positive cells were expressed as a percentage of total counted cells.

### Adipogenesis

Adipose-derived stem cells grown with or without decellularized extracellular matrix were first induced to premature senescence by exposure to 100 μM H_2_O_2_ for 2 h. After washing, the cells were incubated in in complete media for an additional 52 h and then differentiated using Human Mesenchymal Stem Adipogenic Kit (Cyagen Biosciences, Guangzhou, China) according to the manufacturer’s instructions. The kit is based on insulin, dexamethasone, 3-isobutyl-1-methylxanthine, and rosiglitazone. Accumulation of visible neutral lipid vacuoles was confirmed by Oil Red O staining and inverted microscopy after 12–20 days. Adipogenesis was compared among control adipose-derived stem cells and similar cells grown with or without decellularized extracellular matrix and exposed to 100 μM H_2_O_2_.

### Osteogenesis

Adipose-derived stem cells were also differentiated using Human Mesenchymal Stem Osteogenesis Kit (Cyagen Biosciences, Guangzhou, China), which is based on dexamethasone, b-glycerophosphate, and l-ascorbic acid-2-phosphate. After 2–4 weeks of culture, mineralization was confirmed by alizarin red staining. Cells were then cultured in basal media (control) or on decellularized extracellular matrix, treated with or without 100 μM H_2_O_2_, and imaged on an inverted microscope.

### Intracellular ROS

Intracellular reactive oxygen species (ROS) was quantified by flow cytometry to measure the oxidation of cell-permeable DCFH-DA (2′,7′-dichlorodihydrofluorescein diacetate) to fluorescent dichlorofluorescein following the manufacturer’s instructions for Reactive Oxygen Species Assay Kit (Solarbio Science, Beijing, China). In short, 3 × 10^5^ detached cells/well, in quadruplicate (*n* = 4), were labeled for 20 min at 37°C with 10 μmol/l DCFH-DA and then washed twice in PBS. Mean fluorescence intensity was measured using FlowJo software and a BD Biosciences FACSVerse flow cytometer.

### Statistical analysis

All data in the present study were expressed as mean ± standard error of mean (SEM) from at least three independent experiments. Statistical analysis was performed using one-way analysis of variance followed by LSD-t test to investigate each variable for differences among the groups using SPSS 20.0 software. Significance was indicated by a *P*-value of < 0.05.

## Results

### Characterization of adipose-derived stem cells

**Figure 1:**
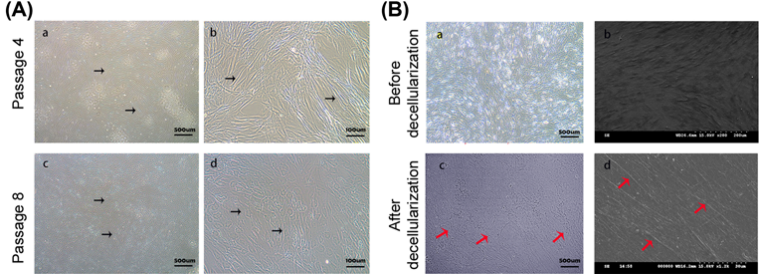
The morphology of adipose-derived stem cells extracellular matrix (**A**) Representative adipose-derived stem cells at passage 4 (early) and 8 (senescent) were imaged at low (a, c) and high (b, d) magnification. (The black arrows represent the adipose-derived stem cells.) (**B**) Extracellular matrix harvested from adipose-derived stem cells before (a, b) and after (c, d) decellularization. (The red arrows represent the visible portion of the extracellular matrix.) Left panels are images from an inverted microscope. Right panels show three-dimensional structures.

Primary adipose-derived stem cells began to extend after 4 h, and grew rapidly after adherence. Cell aggregation reached 70–80% in only 2–3 days. After several generations of purification, cells were uniformly long and spindle-shaped in appearance, and proliferated in colonies that formed orderly swirls (Figure 1A, panels a and b). However, senescence-associated changes were observed after eight generations, including increased cell volume and heterogeneity, flat and oval-shaped morphology, atrophy or even loss of cellular processes, and diminished proliferation (Figure 1A, panels c and d). Hence, adipose-derived stem cells were used in experiments before the eighth passage.

### Morphology of the extracellular matrix

The morphology of the extracellular matrix was visualized by inverted and scanning electron microscopy (Figure 1B). After induction with ascorbic acid, human adipose-derived stem cells abundantly secreted extracellular matrix until only very few cells remained clearly visible (Figure 1B, panels a and b). By imaging on an inverted microscope, we found that the matrix is a fibrous network with tight and organized features, and is cell-free after decellularization (Figure 1B, panel c). High-power electron microscopy also showed that the matrix consists mainly of bulky and staggered collagen fibers and fibronectin of various thickness (Figure 1B, panel d) that form a three-dimensional scaffold.

### Effect of autologous extracellular matrix on proliferation

**Figure 2:**
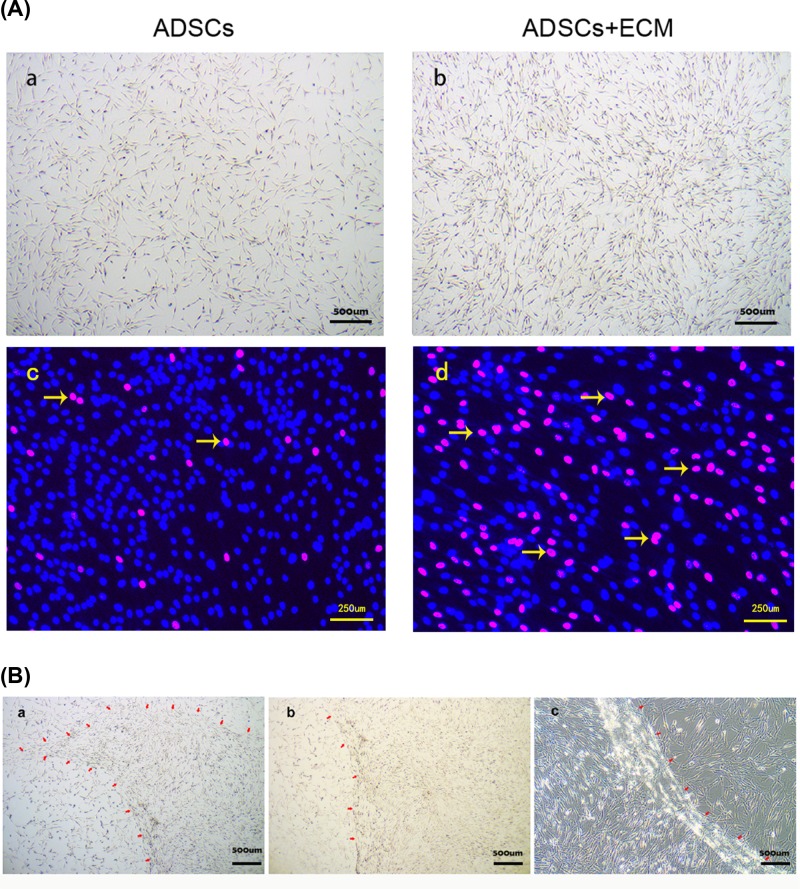
The effect of autologous extracellular matrix on proliferation in adipose-derived stem cells (**A**) Adipose-derived stem cells cultured on decellularized extracellular matrix (b) proliferated more robustly than control cells (a). Edu staining also revealed fewer replicating cells in the decellularized extracellular matrix treated group (c) than in the control group (d). Yellow arrows represent the cells with active DNA synthesis. (**B**) After decellularization in a single plate, we peeled off part of the extracellular matrix with sterile forceps and incubated cells on it. Significant differences in adipose-derived stem cells growing in areas with or without decellularized extracellular matrix in the same plate, as observed at low (a and b) and high (c) magnification.

As shown in Figure 2A (panels a and b), adipose-derived stem cells were more abundant on decellularized extracellular matrix than on culture flasks, and were also more densely packed. Meanwhile, the percentage of areas covered by adherent cells in the decellularized extracellular matrix group (77.3±5.2%) was more than double that in the control group (32.4±3.8%). Furthermore, similar trends were observed on staining with Edu (Figure 2A, panels c and d), indicating more active DNA synthesis in the presence of decellularized extracellular matrix. These differences are clearly observed in a single plate shown in Figure 2B, in which the edges of the extracellular matrix are marked with red arrows to show cells proliferating more extensively on the matrix than beyond it. These results clearly suggest that autologous decellularized extracellular matrix promotes the expansion of adipose-derived stem cells *in vitro*.

### H_2_O_2_-induced aging

**Figure 3:**
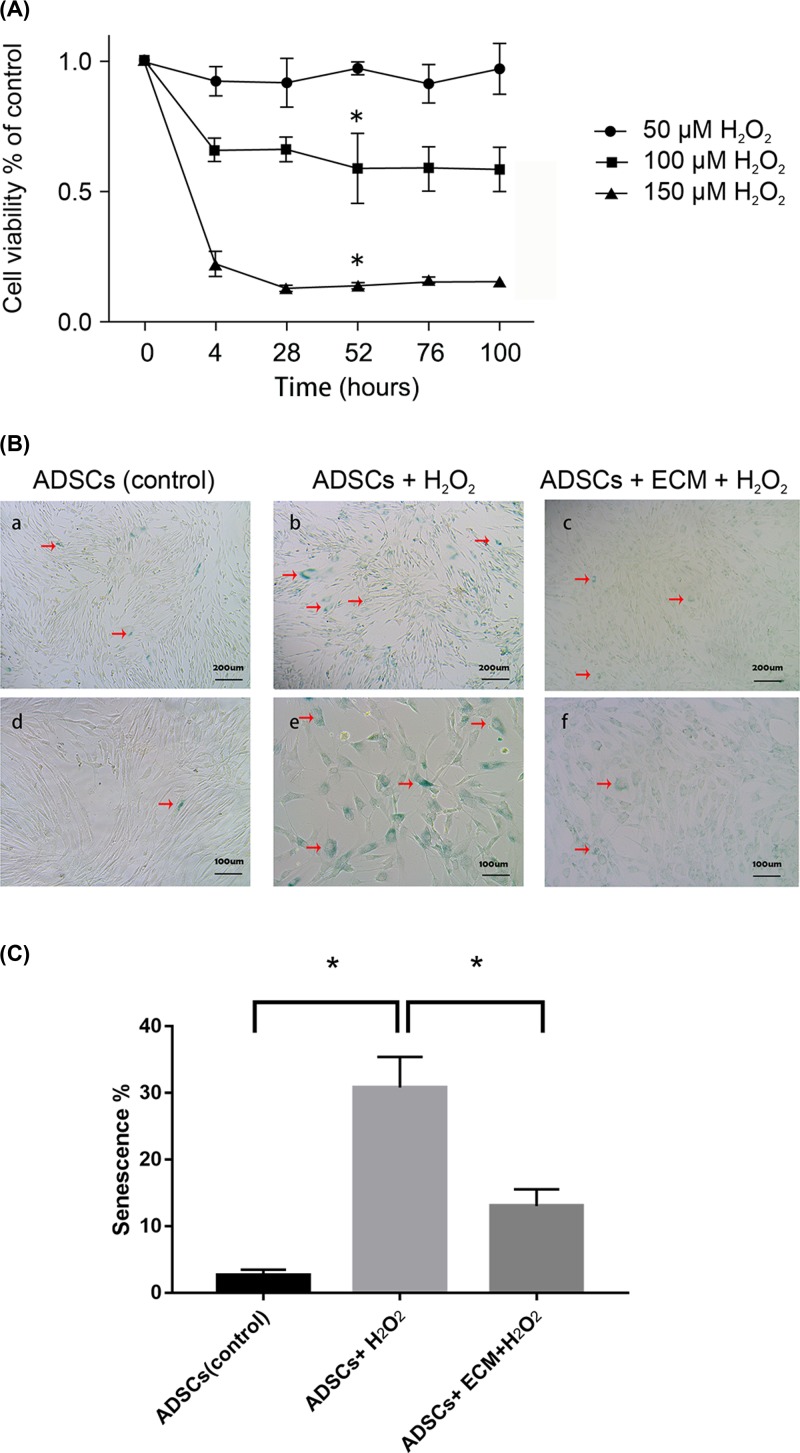
The optimal concentration of H_2_O_2_ to elicit aging and its effect on senescence-associated β-galactosidase with or without decellularized extracellular matrix (**A**) Cell viability was assessed in adipose-derived stem cells treated with different concentrations of H_2_O_2_ to determine the optimal concentration that elicits aging but not apoptosis. Data are mean relative growth rates ± SEM. *, *P* < 0.05, at the time point of 52 h, when 100 μM H_2_O_2_ treated group was compared with control group, 150 μM H_2_O_2_ treated group was compared with control group. (**B**) Effect of senescence-associated β-galactosidase on adipose-derived stem cells treated without (a, b) or with 100 μM H_2_O_2_ for 2 h (c, d), and in cells grown on extracellular matrix post-exposure to H_2_O_2_ (e, f). The red arrows represent the senescent cells which are stained dark green. (**C**) The percentage of senescence-associated β-galactosidase positive cells was calculated on control group, 100 μM H_2_O_2_ treated group and decellularized extracellular matrix plus 100 μM H_2_O_2_ treated group (results are presented as means ± SEM for five replicates per point). *, *P* < 0.05, when 100 μM H_2_O_2_ treated group was compared with control group, decellularized extracellular matrix plus 100 μM H_2_O_2_ treated group was compared with 100 μM H_2_O_2_ treated group.

Adipose-derived stem cells were exposed for 2 h to 50, 100, or 150 μM H_2_O_2_ and assessed by Cell Counting Kit-8 (Dojindo, Japan) after maintaining in complete media for different times. As seen in Figure 3A, cell viability diminished sharply at 150 μM, indicating lethal oxidative stress. In contrast, viability at 50 μM was comparable to that in control cells, as indicated by a growth curve close to 1, indicating minimal oxidative stress. However, cell growth was inhibited 50–60% at 100 μM, although colonies were not completely eliminated. Meanwhile, the curves tended to be flat after 52 h, indicating a stable cell viability post the senescent-related stress condition. Therefore, re-culturing in fresh growth medium for an additional 52 h after induced by 100 μM H_2_O_2_ for 2 h was used in subsequent experiments as needed to stress stem cells.

### Effect of extracellular matrix on senescence-associated β-galactosidase

As cells age but remain viable, gene and protein expression profiles are dramatically altered, with endogenous senescence-associated β-galactosidase accumulating. This effect was observed in cells treated with H_2_O_2_ (Figure 3B, panels b and e) but not in untreated cells (Figure 3B, panels a and d), confirming premature senescence in the former. Strikingly, cells expressing senescence-associated β-galactosidase, which are stained dark green in this assay, were significantly fewer in H_2_O_2_-induced adipose-derived stem cells growing on decellularized extracellular matrix than in similar cells growing in basal media (Figure 3B, panels c and f). Further, the percentage of senescence-associated β-galactosidase positive cells (Figure 3C) confirmed that the decellularized extracellular matrix protected cells *in vitro* against senescence due to oxidative stress.

### Effect of extracellular matrix on adipogenesis and osteogenesis

**Figure 4:**
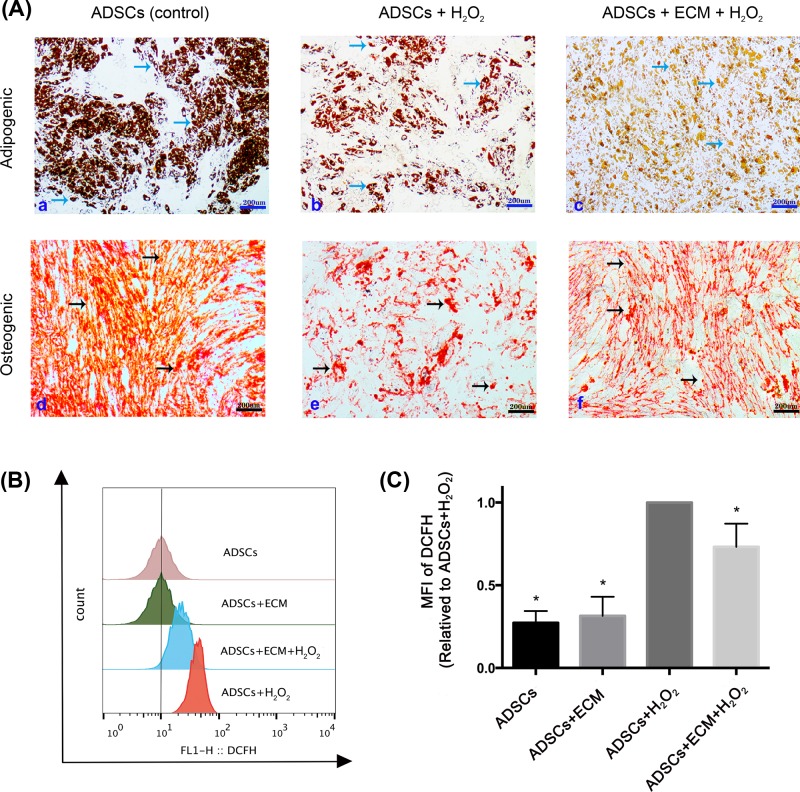
The effect of decellularized extracellular matrix on osteogenesis, adipogenesis, and intracellular reactive oxygen species in adipose-derived stem cells Adipose-derived stem cells were first pretreated to cause premature senescence by exposure to 100 μM H_2_O_2_ for 2 h. After washing, the cells were incubated in complete medium for an additional 52 h and then differentiated into adipocyte and osteocyte. (**A**) There was a decline in lipid droplets and bone matrix formation in H_2_O_2_-induced adipose-derived stem cells (b and e) compared with the control group (a and d). However, the results in the decellularized extracellular matrix treated H_2_O_2_-induced cells (c and f) were comparable to those observed in the controls, which indicated the protection potency of the extracellular matrix in maintaining good abilities of adipogenic and osteogenic when treated with H_2_O_2_. Black arrows represent lipid droplets and blue arrows represent bone matrix. (**B** and **C**) H_2_O_2_ significantly induced accumulation of ROS when cells grown with decellularized extracellular matrix. Data are mean ± SEM of the mean fluorescence intensity (*n* = 4). Statistically significant differences are indicated by * (*P* < 0.05).

Although adipose-derived stem cells appeared to be multipotent and were both adipogenic and osteogenic (Figure 4A, panels a and d), H_2_O_2_-induced cells did not form lipid droplets and bone matrix as profusely, were in poor health, and even lost some stemness (Figure 4A, panels b and e). Strikingly, the decellularized extracellular matrix mitigated the effects of H_2_O_2_ on adipogenicity and osteogenicity (Figure 4A, panels c and f) and preserved stemness comparable to that of control cells.

### Effect of extracellular matrix on intracellular reactive oxygen species

The mean fluorescence intensity from DCFH, a probe of ROS, increased nearly three-fold following exposure to H_2_O_2_, an effect partially reversed by the decellularized extracellular matrix (Figure 4B). This loss of fluorescence was confirmed by a rightward shift in FlowJo histograms (Figure 4C) from H_2_O_2_-induced cells growing on decellularized extracellular matrix in comparison with histograms from similar cells growing without decellularized extracellular matrix.

## Discussion

Strikingly, seeding on extracellular matrix harvested from the adipose-derived stem cells by decellularization clearly reverses many of the effects of H_2_O_2_, and stimulates proliferation *in vitro*. Indeed, even aging adipose-derived stem cells appear to retain excellent multipotency when grown on decellularized extracellular matrix, implying that stemness is preserved. Further, this three-dimensional scaffold consists of complex array of staggered protein fibers indicates that the protective effects of the matrix are probably due to the mechanical properties as mentioned in previous studies [12]. Retention of stemness also implies that the decellularized extracellular matrix provides essential microenvironmental cues, presumably because of deposited growth factors, cytokines, and matrix molecules, which have already been shown to alter the metabolism and biological activities of stromal cells [13].

Generally, cells age in one of two ways. In the first way, cells gradually reach their growth limit and stop dividing *in vitro* following replicative senescence. In the other, stem cells are exposed to dramatic changes in the microenvironment either *in vivo* or *in vitro*, resulting in premature senescence [14,15]. However, replicative senescence develops gradually and very slowly; so, we first sought to establish a model of cell senescence using hydrogen peroxide, a dominant ROS that was previously shown to induce senescence in mesenchymal stem cells [16,17]. In particular, preventing ROS from accumulating is important to maintain stem cells in a resting state, although a burst of such species may conversely be used to promote stem cell differentiation and proliferation. Nevertheless, excessive ROS may also damage lipids, proteins, and nucleic acids, and potentially trigger stem cell apoptosis. Based on preliminary tests, we determined the optimum H_2_O_2_ concentration to induce senescence but not apoptosis. Importantly, adipose-derived stem cells treated with H_2_O_2_ exhibited oxidative stress, aging, and senescence-associated phenomena, but actively proliferated while accumulating senescence-associated β-galactosidase and ROS. However, no other significant physiological differences were observed.

The decellularized extracellular matrix tested in the present study is different from those used in various other stem cell culture systems, because it was naturally synthesized and secreted from autologous seed cells, and thus is rich in collagen, fibronectin, small glycoproteins, and matrix membrane proteins, as observed on scanning electron micrographs and as previously described [18]. Accordingly, stem cells grown on the matrix become embedded and acquire a three-dimensional environment similar to that *in vivo*, which is required for cell growth in that context. Importantly, cells seeded on extracellular matrix exhibited no apparent cytotoxicity, proliferated robustly, and accumulated faster than in standard culture dishes.

Previous studies indicated that cellular senescence depends mainly on p53/p21 and p38 mitogen-activated protein kinase/p16INK4α. Activation of p53 and its target, p21, is associated with DNA damage [19], while p16INK4α deactivates the cell cycle kinases CDK4 and CDK6, both of which accumulate in H_2_O_2_-induced cells that exhibit aging but are diminished in cells protected by the decellularized extracellular matrix [20]. Recently, Zhou et al. [21] demonstrated that SIRT1 signaling also mediates the protective effects of the decellularized extracellular matrix derived from marrow stem cells against premature senescence due to oxidative stress. However, we emphasize that the mechanisms underlying the protective effects of the decellularized extracellular matrix of stem cells are not yet fully understood. In particular, the matrix proteins that confer such protection are unknown, as is the mechanism by which the matrix controls the cellular response to growth factors. Moreover, cell senescence is not only a potential cause of aging but also a way for organisms to inhibit tumors. Therefore, any extracellular matrix used in therapy or to grow cells that are then used in therapy must be verified to not interfere with senescence as a physiological anti-tumor mechanism.

Cellular senescence should be considered a critical issue in cell therapy, primarily because it is induced by long-term culture and a variety of environmental factors. Hence, we anticipate that the anti-aging effects of the decellularized extracellular matrix will prove valuable in the therapeutic applications of adipose-derived stem cells by enhancing cell performance *in vitro* and *in vivo*. We also anticipate that efficient production of the autologous decellularized extracellular matrix may extend the effective life of stem cells *in vitro* while avoiding some of their undesirable properties. Moreover, adipose-derived stem cells used in most studies are suspensions of naked cells that are vulnerable to the immune system, resulting in poor survival after injection, as well as in unexpected stem cell behaviors [22–24]. Therefore, protecting adipose-derived stem cells with the decellularized extracellular matrix may significantly enhance survival and ameliorate cell-assisted lipotransfer, tissue repair, or other clinical applications.

In summary, our study showed that short-term treatment with H_2_O_2_ accelerates senescence in adipose-derived stem cells. However, supplementation with autologous decellularized extracellular matrix reverses this effect, as indicated by fewer cells expressing senescence-associated β-galactosidase, lower levels of ROS, and higher levels of multipotency markers. In addition, the extracellular matrix strongly promotes proliferation, enabling robust cultures. It is possible that adipose-derived stem cells naturally secrete extracellular matrix precisely to prevent aging and regulate proliferation. Thus, the data provide valuable theoretical applicability of autologous decellularized extracellular matrix for anti-aging and regenerative treatments.

## Abbreviations

DCFH-DA: 2′,7′-dichlorodihydrofluorescein diacetate
PBS: phosphate buffer saline
ROS: reactive oxygen species
SEM: standard error of mean
